# Smart-Temporary-Film-Based Local-Delivery System with Controllable Drug-Release Behavior

**DOI:** 10.3390/gels8120773

**Published:** 2022-11-27

**Authors:** Denghang Xie, Huiwen Wang, Cheng Yin, Mengxia Peng, Haiyong Ao, Jian Hu, Yizao Wan, Quanchao Zhang

**Affiliations:** 1 Jiangxi Key Laboratory of Nanobiomaterials, Institute of Advanced Materials, East China Jiaotong University, Nanchang 330013, China; 2 School of Materials Science and Engineering, East China Jiaotong University, Nanchang 330013, China; 3 School of Materials Science and Engineering, Tianjin University, Tianjin 300350, China

**Keywords:** smart, temporary film, microgels, controllable drug release, local delivery system

## Abstract

The development of a simple local drug-delivery system that exhibits the advantages of macro- and microscale carriers with controllable drug-release behavior is still highly desired. Herein, in this work, a smart temporary film was prepared from doxorubicin (DOX)-loaded shape-memory microgels via a simple hot-compression programming method. The temporary film showed a very smooth surface and easy handing, as well as macroscopy mechanical properties, which could disintegrate into the microgels with heating at 45 °C. In this case, the temporary film showed a controllable DOX release behavior when compared with the microgels, which could release the DOX on demand. Consequently, the temporary film exhibited weaker cytotoxicity to normal cells and a much longer antitumor capability, as well as a higher drug-utilization efficiency when compared with microgels. Therefore, the smart temporary film has high potential as a candidate for use as a local drug-delivery system.

## 1. Introduction

Local tumor recurrence after surgery is still a major clinical challenge for most cancers because the traditional chemotherapy is limited with the low efficiency of delivering the drugs specifically to the tumor site at therapeutic concentrations and the ensuing toxicity to healthy tissues [1,2,3]. To overcome these limitations, the local delivery system has been developed [4,5,6], in which the drug-delivery system is directly implanted at the site of resection of the primary tumor and can release drugs locally in the extracellular fluid. Thus, it can enhance the dosage at the targeted site and reduce side effects toward other organs and tissues [7]. Nevertheless, high doses of the toxic drug may cause inflammation. Therefore, the local drug-delivery system with controllable drug-release capability is highly desired.

Two main approaches of programmable and on-demand local delivery systems were applied to trigger the drug-release behavior in response to internal or external stimuli for the local drug-delivery system [8,9,10,11,12,13]. For example, Gu et al. [13] prepared a scaffold that consists of a reactive oxygen species (ROS)-degradable hydrogel that releases therapeutics in a programmed manner within the tumor microenvironment, which contained abundant ROS. It was found that the scaffold elicited an immunogenic tumor phenotype and promoted an immune-mediated tumor regression in the tumor-bearing mice, with prevention of tumor recurrence after the primary resection. Kubota et al. reported the drug-model-encapsulating calcium alginate hydrogel microbeads containing tungsten particles with high acoustic impedance, which can achieve the on-demand drug-release behavior by using ultrasound at 20 kHz [14]. All the above works showed the controllable drug-release capability in different manners, which highly depended on the type of the drug carrier. Generally, macroscale (wafers, hydrogels, foams, and fibers) and microscale (micro/nanotherapeutics) drug carriers are applied for the local drug delivery [15]. The macroscale carriers are easy for handing and manipulation, as well as fixation at the site, while the microscale carriers exhibit a more controllable drug behavior because of their special shape and high surface area. There is a huge gap between the macro- and microscale drug carriers. If a carrier can build a “bridge” between the two scaled size carriers, it would exhibit the advantages of macro- and microscale carriers, which is rarely reported to the best of our knowledge. 

In this regard, the shape-memory polymer, a smart material that can be programmed into various temporary shapes and then can recover to the initial shape with external stimuli [16,17,18,19], has high potential for preparing the local drug-delivery system. The biggest feature of this smart material is that it can follow a preset route to achieve the shape changes under external stimuli. If the shape-memory polymer is prepared into microparticles, due to the small size, the shape change in the microparticles can significantly change the specific surface area, which makes it an ideal drug-carrier material [20]. What is more, in the previous work, a temporary film was prepared from the shape-memory microparticles in a very simple way (hot-compression programming method), which can recover into microparticles again [21]. In this context, we assume that such a temporary film can be used as the local drug-delivery system as it is easy to handle and has macroscopic mechanization when compared with microparticles, which can be easily fixed at the site of treatment (e.g., by suturing) and disintegrate on demand to microparticles, facilitating the drug release. Additionally, the huge surface change from temporary film to microparticles with external stimuli, together with the surface-area change in the microparticles themselves, can achieve the required controllable drug-release behavior.

**Figure 1:**
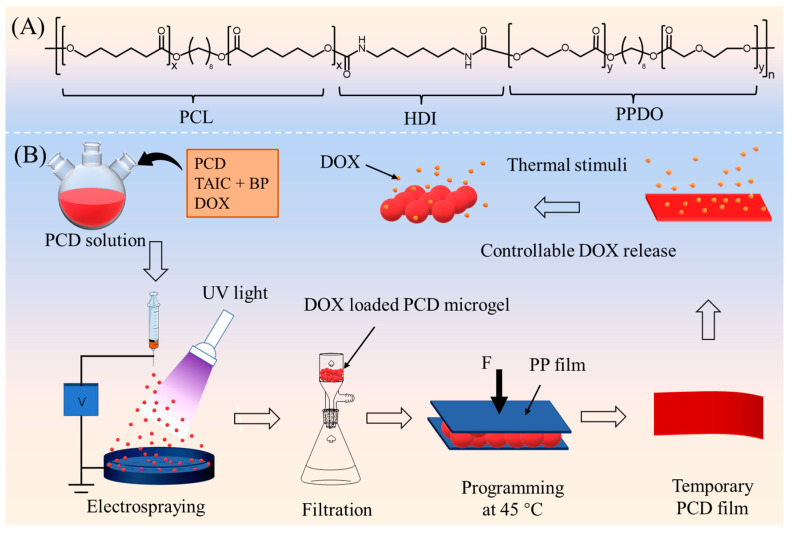
The chemical structure of PCD (**A**) and the scheme of the temporary film prepared from the electrosprayed shape-memory microgels (**B**).

Herein, in this work, a multiblock copolymer of polycaprolactone-poly (*p*-dioxanone) with good shape-memory function was selected as the raw shape-memory polymer and the doxorubicin (DOX) was used as the model drug. Using electrospraying, the microgels were first prepared, and then the temporary film was prepared from electrosprayed shape-memory microgels by a simple hot-compression programming method (Figure 1, for details see Section 4.1 and Section 4.2). Then, the morphologies, thermal and crystal properties, drug-release behavior, cytocompatibility, blood compatibility, and antitumor capability of the temporary film were investigated. 

## 2. Results and Discussion

### 2.1. Morphologies

**Figure 2:**
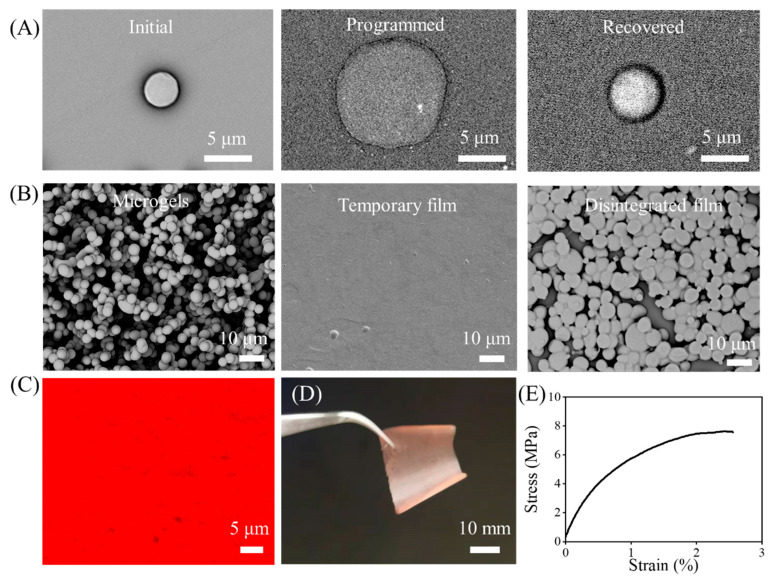
(**A**) SEM images of the initial, programmed, and recovered single microgels. (**B**) SEM images of the microgels with the high deposition density, temporary film, and the disintegrated film. (**C**) CLSM image showing the DOX distribution. (**D**) Digital photo of the temporary film. (**E**) Tensile test of the temporary film.

In this work, the PCD microgels were prepared via electrospraying together with the in situ UV cross-linking. The diameter of the microgels was 3.6 ± 0.7 μm. Using the method described in the previous work [21], the shape-memory effect of the single microgel was investigated first. PCD is a multiblock copolymer comprising two different crystalline domains, whereby PPDO crystallites act as net points, determining its permanent shape, while the PCL domains act as reversible physical cross-links that can be utilized to trigger a shape-memory effect. In this context, the PCD microgels will exhibit the shape-memory effect under the thermal stimulus. As seen in Figure 2A, the diameter of the initial microgel was about 3.6 μm. After the programming at the optimal parameters (45 °C, 1.5 MPa, 10 min), a disk-shaped microgel with the diameter of about 11.8 μm was achieved. The diameter change ratio was about 328%. When heated at 45 °C, the disk shape can recover to its particle shape but cannot completely recover into its initial shape. The shape recovery (diameter) ratio was about 80 ± 5%. Then, the microgels were uniformly deposited onto a PP film surface with high deposition density, which could be transformed into a temporary film using the same programming parameters as that of the single microgel by utilizing its shape-memory effect. Such temporary film could be switched the disintegrated film when heated at 45 °C (Figure 2B). Because the DOX is highly fluorescent, a CLSM image was applied to determine the distribution of the DOX in the temporary film. As seen in Figure 2C, the uniform red fluorescence of DOX could be found, indicating the uniform dispersion of DOX in the film. Figure 2D showed the temporary film was a free-handing thin film with the color of light red caused by the incorporation of DOX, which meant that such film can be easily fixed at the tumor site by suturing or other methods. The thickness of the film was about 5 μm. The temporary film also exhibited the macroscope mechanical properties. As shown in Figure 2E, a typical tensile strain–stress curve could be obtained for the temporary film. It showed a tensile strength of 7.5 ± 0.8 MPa, a Young’s modulus of 426.3 ± 40.0 MPa, and an elongation at break of 2.6 ± 0.3%, respectively. Compared with the reported solid electrospun PCL-PPDO multiblock copolymer fibrous mats (a tensile strength of 4.6 ± 0.7 MPa, a Young’s modulus of 3.3 ± 0.3 MPa, and an elongation at break of 800 ± 40%) [22], the temporary film showed the higher tensile strength and Young’s modulus, as well as the much lower elongation at break. We attributed those differences on the one hand side to the microthickness of the film, and on the other hand side, to the fact that the temporary film was generated via high plastic deformation of the microgel at processing temperatures closely above *T*_m,PCL_ but significantly below *T*_m,PPDO_ (for details see Section 2.2).

**Figure 3:**
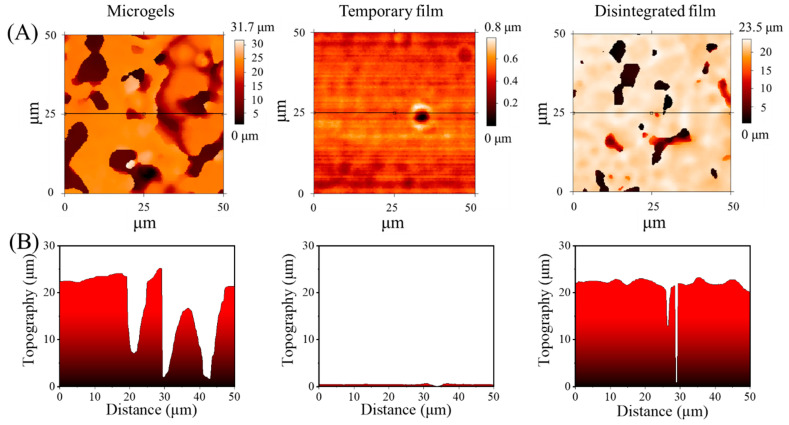
Representative surface profile (**A**) and height profile (**B**) of microgels with high deposition density, temporary film, and the disintegrated film obtained by optical profilometry.

The roughness of the microgels with high deposition density, temporary film, and the disintegrated film were investigated by an optical profilometry. As seen in Figure 3A, the rich colors relating to the depth ranged from 0 to 31.7 μm and could be found for the surface morphology of microgels with high deposition density. In contrast, the temporary film showed a much more uniform color representing the depth that ranged from 0 to 0.8 μm, indicating the surface was very smooth. After the recovery procedure, the disintegrated film showed colors representing the depth that ranged from 0 to 23.5 μm, suggesting that the temporary film cannot be fully disintegrated into the initial microgels, which agreed well with the SEM results. In this context, the root–mean–squared surface roughness of the microgels with high deposition density, temporary film, and disintegrated film was 3.1, 0.1, and 2.3 µm, respectively (Figure 3B). The roughness of the temporary film was similar to that previously reported for the PCL-PPDO multiblock copolymer temporary film (0.4 µm) [21].

### 2.2. Thermal and Crystal Properties

**Figure 4:**
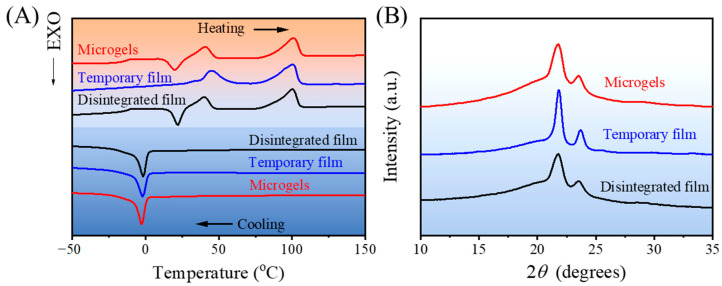
DSC curves (**A**) and XRD patterns (**B**) of microgels, the temporary film, and the disintegrated film.

The thermal properties of the microgels, temporary film, and disintegrated film were analyzed via DSC. As displayed in Figure 4A, two well-separated melting temperatures related to the crystalline PCL (*T*_m,PCL_) and PPDO (*T*_m,PPDO_) domains could be observed in the DSC heating curves for all the samples. The melting peak temperatures were determined for the microgels at *T*_m,PCL_ = 40 °C and *T*_m,PPDO_ = 100 °C, which were identical with the results of the disintegrated film (*T*_m,PCL_ = 40 °C and *T*_m,PPDO_ = 100 °C). Both the microgels and disintegrated film showed the cold crystallization peak at around 21 °C, which was associated with the slow crystallization rate of the PPDO domains [22]. In contrast, the temporary film showed a higher *T*_m,PCL_ = 45 °C and a same *T*_m,PPDO_ = 100 °C when compared with microgels and disintegrated film. The higher *T*_m,PCL_ for temporary film could be due to the stress induced crystallization during the programming (hot compression) [23]. What is more, no cold crystallization peak was found from the DSC curve of temporary film, which could be also due to the completely crystallization of PPDO domains during the programming. From the cooling curves, all the samples showed the crystallization peaks at 2 °C. The relative degree of crystallinity of the microgels was almost identical to that of the disintegrated film with $\chi_{C,\mathrm{PCL}}$ ≈ 32% and $\chi_{C,\mathrm{PPDO}}$ ≈ 48%. For the temporary film, both degrees of crystallinity of $\chi_{C,\mathrm{PCL}}$ = 41% and $\chi_{C,\mathrm{PPDO}}$ = 50% could be determined. It showed that the programming could increase the crystallization degree, which agreed with the reported result [24].

The same trend for their degree of crystallinity can be found from the XRD results. As seen in Figure 4B, all the samples showed quite similar peaks at 2*θ* of 21.8° and 23.5°. PPDO exhibited the characteristic diffraction peaks at 2*θ* of 21.9° and 23.7°, which attribute to the reflection of (210) and (020) [25,26], while PCL shows two characteristic peaks at 2*θ* of 21.2° and 23.5°, which correspond to the (110) and (200) crystallographic planes of the PCL crystalline structure [18]. Therefore, the diffraction peaks for all the samples in this work should be the mixture of the characteristic peaks of PPDO and PCL domains. The crystallization degrees of microgels, temporary film, and disintegrated film were 20%, 24%, and 20%, respectively.

### 2.3. Drug-Release Behavior

**Figure 5:**
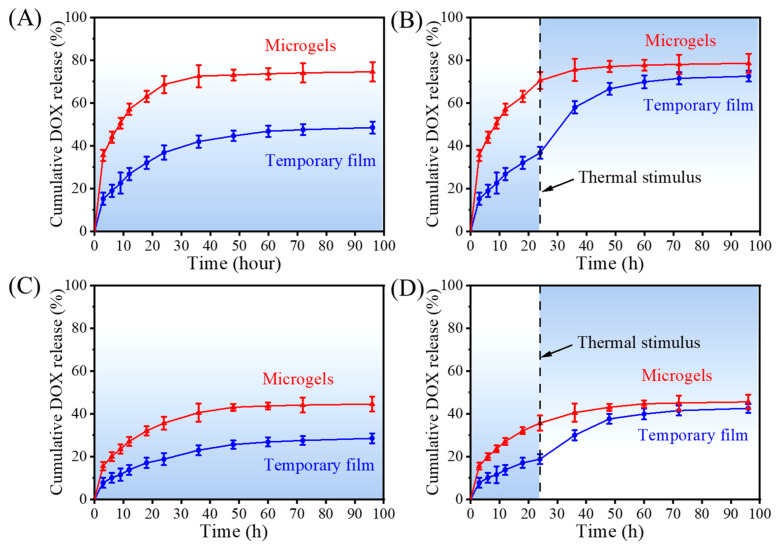
Drug-release profiles of DOX for the microgels and temporary film at pH = 5.6 (**A**,**B**) and at pH = 7.4 (**C**,**D**) without the thermal stimulus (**A**,**C**) and with the thermal stimulus for 10 min after the initial 24 h drug release (**B**,**D**).

Based on the acidic environment of the tumor [27], the drug-release behavior was first examined under the acidic (pH = 5.6) conditions. As seen in Figure 5A, an initial burst release of DOX could be found for the microgels, which is caused by the diffusion of the free drugs from the microgels into the medium. The same release behavior could also be found for other particles and electrospun fibers because of the high surface area [28,29]. The release equilibrium was achieved at around 24 h, with the cumulative release amount of 68.6 ± 4.0%. For the temporary film, even though its surface area was much smaller than that of microgels, a weaker initial burst release of DOX could still be found at the beginning, and the cumulative release amount was about 36.8 ± 3.3% after 24 h release. At the same time, we give the thermal stimulus for both the samples after the initial 24 h release for 10 min at 45 °C. It can be clearly seen that the release rate of the temporary film increased when compared that without thermal stimulus. A second step of the release could be found. The release equilibrium was achieved at around 60 h, with the cumulative release amount of 69.9 ± 3.0%. In contrast, there was only a slight increase (70.5 to 77.7%) in the microgels (Figure 5B). In contrast, the drug-release behavior was then examined at pH = 7.4. As seen in Figure 5C,D, they showed quite similar release profiles as those at pH = 5.6. However, they all showed slower DOX release rates and lower release amounts than those at pH = 7.4, which agrees with the reported results [29,30]. The reason could be that DOX is protonated under acidic conditions. The protonation increases its water solubility and is beneficial for drug release in an acidic tumor environment [31]. In a word, the temporary film showed the controllable DOX release behavior, which can release the DOX on demand. What is more, the releases of DOX at pH = 5.6 and pH = 7.4 from the microgels and temporary film were fitted with the Korsmeyer–Peppas model, which all showed a linear relationship with R^2^ values of 0.99. The value of *n* was calculated to evaluate the release mechanism of the DOX, whereby *n* ≤ 0.45 suggests the release pattern as being Fickian diffusion [32]. During this study, the values of *n* were obtained as 0.30 and 0.43 for microgels and temporary film at pH = 5.6, respectively, while they were 0.39 and 0.44 at pH = 7.4, respectively. Therefore, it was clear that the release of DOX from the microgels and temporary film followed the diffusion-controlled phenomena.

### 2.4. Cytotoxicity and Blood Compatibility

**Figure 6:**
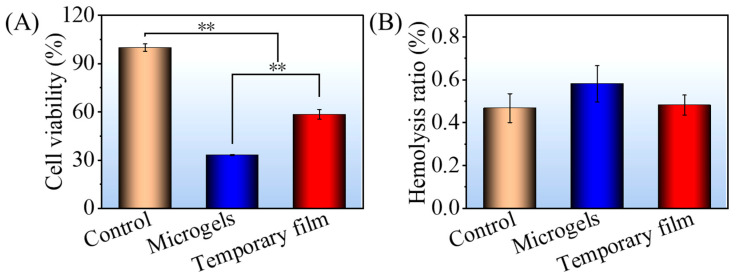
Cytotoxicity of NIH3T3 cells (**A**) and hemolysis rate (**B**) of PCD film (control), microgels, and temporary film. Significance was defined as ** *p* < 0.01, *n* = 5.

Here, the NIH3T3 cells were cocultured on PCD film (control), microgels, and temporary film to assess the cytotoxicity. As seen in Figure 6A, the viability values of *NIH3T3* cells cocultured with the control, microgels, and temporary film were 100%, 33%, and 59%, respectively, indicating that DOX-loaded samples showed the cytotoxicity. However, when compared to microgels, the temporary film showed a much lower cytotoxicity. 

The hemolysis rate is an important indicator of blood compatibility. A hemolysis rate below a threshold of 5% means minimal harm to red blood cells [33]. Figure 6B shows the hemolysis rate of blood on PCD film (control), microgels, and temporary film. The hemolysis rates of all three samples were far below 5%, indicating their good blood compatibility.

### 2.5. Antitumor Activities

**Figure 7:**
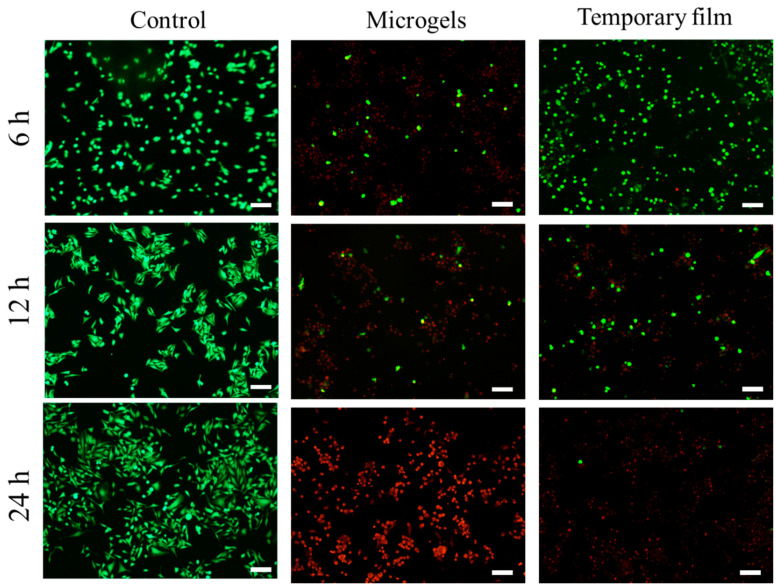
Live (green)/dead (red) staining Hela cells for PCD film (control), microgels, and temporary film samples. Scale bar: 100 μm.

As a typical and broad-spectrum chemical drug, doxorubicin (DOX) can damage DNA, impair its synthesis, and generate oxygen-free radicals, causing cell death [34,35,36]. In this work, the antitumor activities were first determined by live/dead-cell staining (Figure 7). The Hela cell can spread and proliferate well after incubation with the PCD film, suggesting that this film does not possess antitumor properties. However, the number of live cells (green) for the microgels and temporary film showed a continuous decrease with time and more dead cells (red) could be found. When compared with the microgels, the temporary film showed a weaker antitumor activity at the times of 6 and 12 h. The reason could be that, at the same time, more DOX was released from the microparticles. However, after coculture for 24 h, almost all the cells were killed for both samples. 

**Figure 8:**
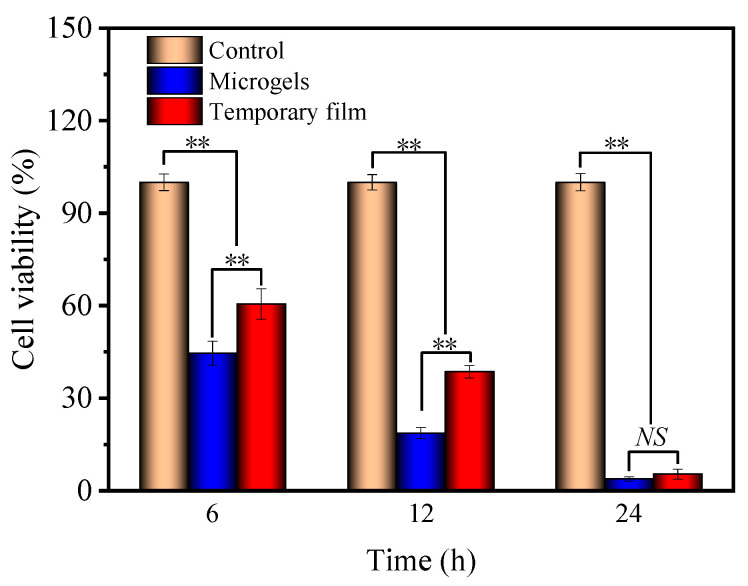
Evaluation of cytotoxic effects (antitumor activities) of PCD film (control), microgels, and temporary film on Hela cells at the culture times of 6, 12, and 24 h. Significance was defined as ** *p* < 0.01, *n* = 5.

The CCK8 assay was further performed to evaluate the antitumor activities of the microgels and temporary film. As seen in Figure 8, for the control, the cell viability was kept at 100% at the different culture times. A much lower cell viability for microgels and temporary film can be found, which further decreased with the culture time. The cell viability between the microparticles and temporary film showed significant differences (*p* < 0.01) at 6 and 12 h. After the 24 h culture, the cell viability was near to zero, and no significant differences were found for the cell viability of the microgels, which is consistent with the results of the live/dead-cell staining.

**Figure 9:**
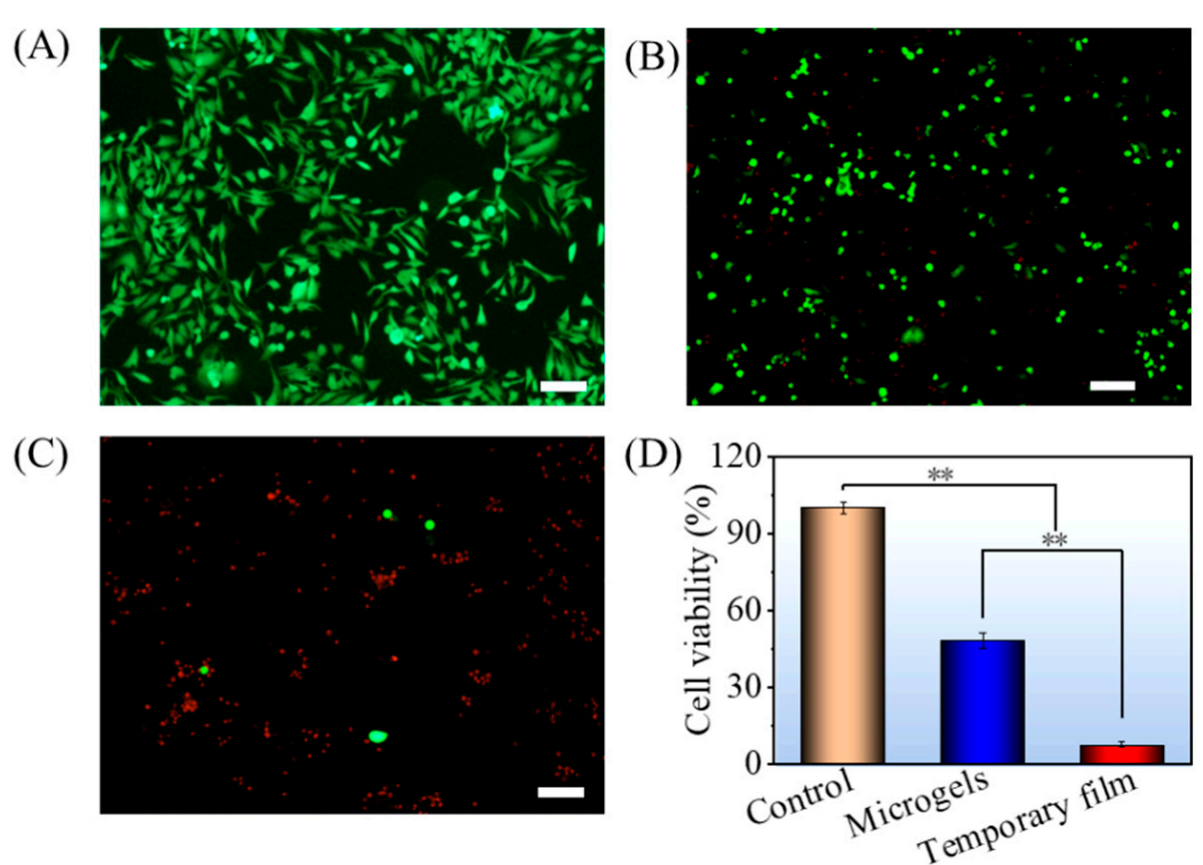
Antitumor activities of the PCD film (control), microgels, and temporary film after incubation for 24 h. The microgels and temporary film were heated at 45 °C for 10 min after the initial 24 h drug release. (**A**–**C**) Live (green)/dead (red)-stained Hela cells for control (**A**), microgels (**B**), and temporary film (**C**). Scale bar: 100 μm. (**D**) Cytotoxicity to Hela cells. Significance was defined as ** *p* < 0.01, *n* = 5.

DOX has been widely used in the treatment of various cancers. However, DOX is also associated with serious side effects, such as increased lipid oxidation, inhibition of nucleic acid and protein synthesis, abnormal calcium ion regulation, tumor cell resistance, and cardiotoxicity [37]. Therefore, it is very important to release the DOX in a controllable manner, which can increase the drug utilization and, at the same time, reduce the side effects on normal tissues. Therefore, we further tested the antitumor activities of the microgels and temporary film after the initial 24 h drug release. All the samples were under the thermal stimulus (45 °C for 10 min) before the cell culture. As seen in Figure 9A, the control group (PCD film) still showed a good cell viability of about 100% after the 24 h culture. The microgels showed a weak tumor inhibiting ability when compared to the temporary film (Figure 9B,C), as most of the drug was released at the beginning 24 h for the microgels. Even under the thermal stimulus, only slightly more DOX was released, which was not enough to kill the cancer cells. The CCK-8 assay results further approved this result. The cell viabilities of the control, microgel, and temporary film were about 100%, 50%, and 7%, respectively (Figure 9D). The results further showed that the temporary film with the controllable DOX release behavior exhibited a higher drug utilization effect and reduced the side effects when compared to the microgels.

## 3. Conclusions

In this work, the temporary film with the thickness of 5 μm was prepared via the electrosprayed DOX-loaded microgels by utilizing the shape-memory effect of the microgels. The temporary film was easy to handle and showed macroscopic mechanical properties, and could disintegrate into the microgels under thermal stimulus. The roughness test showed that the temporary film displayed a very smooth surface. The DSC results showed that the temporary film showed a higher crystallization degree, which could be further approved by XRD results. The temporary film showed a controllable DOX release behavior when compared with the microgels, which can release DOX on demand. In this case, the temporary film showed a much higher drug utilization efficiency and a longer antitumor activity. What is more, the temporary film exhibited weaker cytotoxicity to normal cells when compared with the microgels and showed a good hemolysis rate. Therefore, this work developed a simple smart temporary film with controllable drug-release behavior, which exhibited both the advantages of macroscale and microscale carriers, and thus has high potential for use as a local drug-delivery system.

## 4. Materials and Methods

### 4.1. Materials

A multiblock copolymer of polycaprolactone–poly(*p*-dioxanone) (PCL-PPDO, named as PCD, number-average molecular weight (*M*_n_) of 60,000 g/mol determined by gel-permeation chromatography with polystyrene standards) was used as the raw material (Figure 1A), which was synthesized via the previous procedure [21]. Briefly, PCD was prepared by a cocondensation method from a precursor oligo(*ε*-caprolactone) diol (*M*_n_ = 2000 g/mol, Sigma-Aldrich, Munich, Germany) and oligo (*p*-dioxanone) diol (*M*_n_ = 5000 g/mol) using hexamethylene diisocyanate (HDI, AR, >99%, Macklin Biochemical Co., Ltd., Shanghai, China) as junction unit. Oligo (*p*-dioxanone) diol was prepared by ring-opening polymerization [38]. Benzophenone (BP, AR, 99%) and triallyl isocyanurate (TAIC, AR, ≥99%) were obtained from Shandong Xiya Chemical Ltd., Linyi, China. The solvents of hexafluoroisopropanol (HFIP, AR) and dichloromethane (DCM, AR) were obtained from Sigma-Aldrich (Munich, Germany). DOX (medical grade) was purchased from Meilun Biotechnology Co., Ltd., Dalian, China. Mouse fibroblast NIH3T3 cells were provided by Shanghai Ninth People’s Hospital, Shanghai, China. Hela cells (CL-0101, human cervical carcinoma cells) were purchased from Wuhan Pricella Life Technology Co., Ltd., Wuhan, China. Dulbecco’s modified eagle medium (DMEM, BR), phosphate buffer solution (PBS, BR), and fetal bovine serum (FBS, BR) were obtained from Hyclone Corporation, Shanghai, China. Cell counting Kit-8 (CCK-8, BR) was obtained from Solarbio Technology Co., Ltd., Beijing, China. Fresh anticoagulant rabbit blood was obtained from G-Clone Biotechnology Co., Ltd., Beijing, China. Fluorescein diacetate (FDA, BR), propidium iodide (PI, BR), and other reagents were purchased from Shanghai Aladdin Reagents Co., Ltd., Shanghai, China.

### 4.2. Preparation of Temporary Film

The PCD microgels with 1 wt% DOX, 1 wt% BP, and 1 wt% TAIC were prepared by electrospraying the 2 wt% PCD/HFIP-DCM solution (the volume ratio of HFIP and DCM was 7/3) onto the ice-water receiving bath at room temperature under the ultraviolet (UV) light, with a wavelength of 365 nm. The main processing parameters were as follows: the needle-to-ice-water-receiving-bath distance was 30 cm, the voltage was 10 kV, and the solution flow rate was 2 mL/h. Afterward, the microgels were collected by filtration and then freeze-dried using a freeze drier (FD-1C-50, Biocool, Beijing, China). About 50 mg of microgels was uniformly dispersed on a polypropylene (PP) film, which was compressed at 45 °C under the pressure of 1.5 MPa. After cooling to 10 °C for 10 min, the temporary film, which can be disintegrated into microgels and thus be used as a controllable drug-release system, was obtained (Figure 1B).

### 4.3. Characterization

The morphologies of electrosprayed microgels, temporary film, and the disintegrated film were characterized by scanning electron microscopy (SEM; FEI Nano 430, FEI Company, Hillsboro, USA). To observe the shape changes in the microgel at different temperatures, an auxiliary high-vacuum heating platform was used. For analysis of the average microgel size, 100 particles were measured by using ImageJ. Their surface roughness (*R*a) was determined by an optical profilometry (MicoProf200, Fries Research & Technology GmbH, Bergisch Gladbach, Germany). The surface profiles were measured by scanning an area of 50 × 50 μm in the center of the sample. The distribution of DOX in the temporary film was examined by confocal laser-scanning microscopy (CLSM; Leica TCS SP8, Heidelberg, Germany). The tensile test of the temporary film was measured by a microelectromagnetic fatigue-testing machine (MUF-1050, Tianjin Care Measure & Control Co., Ltd., Tianjin, China) at room temperature, with the strain rate of 5 mm/min. The size of the test samples was 10 × 4 mm, with a thickness of 5 μm. At least three measurements were conducted. The thermal properties of the electrosprayed microgels, temporary film, and the disintegrated film were explored by differential scanning calorimetry (DSC 3, Mettler Tolledo Co., Ltd., Zurich, Switzerland). All experiments were conducted in the temperature range from −60 to 160 °C, with a constant heating and cooling rate of 10 °C/min. Their crystalline structure was characterized by X-ray diffraction (XRD, D8 ADVANCE, Bruker-Tech Co., Ltd., Karlsruhe, Germany).

### 4.4. Drug-Release Behavior

To determine the drug-release behaviors of temporary film, the samples (about 10 mg) with the size 10 mm × 10 mm × 0.005 mm were immersed in 3 mL of PBS under gentle shaking at 100 rpm at 37 °C. An aliquot of release medium (0.2 mL) was collected at predetermined time intervals for analysis. The released DOX was quantified with a Nanodrop 2000 spectrophotometer (NanoDrop, Wilmington, DE, USA) by measuring the absorbance at 480 nm. Further, the cumulative release of the drug was determined based on the standard curve obtained from pure DOX. The same-weight microgels (10 mg) used to form the temporary film were used as the control. To investigate the influence of the thermal stimulus on the drug-release behavior of the temporary film and microgels, the above same procedure was carried out, except for the thermal stimulus (heat at 45 °C for 10 min), after the release time of 24 h for both samples. The Korsmeyer–Peppas model was used to analyze the drug-release kinetics obtained from the microgels and temporary film by using the equation *M*_t_/*M*_α_ = *K*t^n^, where *M*_t_ and *M*_α_ are the amounts of drug released at time t and infinite time (maximum release amount), respectively, while *K* represents the release rate constant and n is the release exponent (diffusional coefficient) [32].

### 4.5. Cytocompatibility Test

To evaluate the cytotoxicity of the microgels and temporary film, the NIH3T3 cells were seeded on the samples at a density of 1.5 × 10^4^ cells per well. The incubation was carried out in DMEM supplemented with 10% FBS and 1% penicillin–streptomycin solution for 24 h at 37 °C in a 5% CO_2_ environment. After completion of incubation, a routine CCK-8 assay was added to each well in darkness and the cells were conducted to assess the proliferation by measuring the absorbance at 450 nm using a microplate reader (iMark, Bio Rad, Hercules, CA, USA). 

### 4.6. Assessment of Blood Compatibility

To test hemolysis rate, PBS-diluted fresh anticoagulant rabbit blood was prepared at a volume ratio of rabbit blood/PBS = 4/5. The diluted blood was dropped onto microgels, temporary film, and hot-compression film (as control) and incubated at 37 °C for 2 h. Subsequently, the diluted blood was centrifuged at 3000 rpm for 5 min and the supernatant was collected for the determination of OD at 540 nm with NanoDrop (2000, Thermo Fisher Co., Ltd., Waltham, MA, USA). The hemolysis rate (*HR*) was calculated following a previous report [39].

### 4.7. Antitumor Experiments

To evaluate the antitumor activities of the microgels and temporary film, the Hela cells were seeded on the samples at a density of 1 × 10^4^ cells per well and then were incubated in 96-well plates filled with DMEM with 10% FBS and 1% penicillin–streptomycin solution. After 24 h incubation, the microgels, the temporary film, and the hot-compression PCD film (as control) were added into each well and incubated for 6, 12, and 24 h at 37 °C in a 5% CO_2_ environment. After completion of incubation, a CCK assay was added to each well in darkness and the cells were conducted to assess the proliferation by measuring the absorbance at 450 nm using the above microplate reader (iMark, Bio Rad, Hercules, CA, USA). 

Fluorescent cell images were obtained using live/dead assay. After incubation for 6, 12, and 24 h under the aforementioned conditions and being washed twice with PBS, cells on the samples were stained for 30 s using FDA/PI and then observed using a fluorescence-inverted microscope (TS2, Nikon, Tokyo, Japan) at 490 nm and 550 nm to determine the morphology of live and dead cells on the samples, respectively. 

At the same time, after the 24 h drug release at PBS, the microgels and temporary film were then used to determine their antitumor activities. The samples were first under a heat stimulus (45 °C) for 10 min. Then, repeating the above procedure, the CCK-8 values and fluorescent cell images were achieved after the 24 h incubation.

### 4.8. Statistical Analysis

Each experiment was conducted in quadruplicate. The data were presented as means ± standard deviations. The one-way analysis of variance (ANOVA) coupled with Fisher’s least-significant difference (LSD) post hoc test were used to determine the level of significance. A *p* value less than 0.05 was considered significant.

## Data Availability

Not applicable.
